# *Chlamydia pneumoniae*-Specific IgE Is Prevalent in Asthma and Is Associated with Disease Severity

**DOI:** 10.1371/journal.pone.0035945

**Published:** 2012-04-24

**Authors:** David L. Hahn, Allison Schure, Katir Patel, Tawanna Childs, Eduard Drizik, Wilmore Webley

**Affiliations:** 1 Departments of Family Medicine, University of Wisconsin School of Medicine and Public Health, and Dean Clinic, Madison, Wisconsin, United States of America; 2 Department of Microbiology, University of Massachusetts, Amherst, Massachusetts, United States of America; University of California Merced, United States of America

## Abstract

**Background:**

Several *Chlamydia pneumoniae* (Cp) biomarkers have been associated with asthma but Cp-specific IgE (Cp IgE) has not been investigated extensively. Our objective was to investigate Cp IgE in community adult asthma patients.

**Methods:**

(1) Prevalence of Cp IgE (measured by immunoblotting) and Cp DNA (by polymerase chain reaction) in peripheral blood, and biomarker associations with asthma severity. (2) Case-control studies of Cp IgE association with asthma using healthy blood donor (study 1) and non-asthmatic clinic patient (study 2) controls.

**Results:**

Of 66 asthma subjects (mean age 40.9 years, range 5–75, 59% male, 45% ever-smokers) 33 (50%) were Cp IgE positive and 16 (24%) were Cp DNA positive (P = 0.001 for association of Cp IgE and DNA). Cp IgE was detected in 21% of mild intermittent asthma v 79% of severe persistent asthma (test for trend over severity categories, P = 0.002). Cp IgE detection was significantly (P = 0.001) associated with asthma when compared to healthy blood donor controls but not when compared to clinic controls.

**Conclusions:**

Half of this sample of community asthma patients had detectable IgE against *C. pneumoniae*. Cp IgE was strongly and positively associated with asthma severity and with asthma when healthy blood donor controls were used. These results support the inclusion of Cp IgE as a biomarker in future studies of infectious contributions to asthma pathogenesis.

## Introduction

“Bacterial allergy" was once thought to be a mechanism linking respiratory bacterial infections and asthma symptoms [1]. Currently, viral infections are widely acknowledged as precipitants of asthma exacerbations, and may be involved in the natural history of asthma, as reviewed elsewhere [2]. Virus-specific IgE (e.g. against RSV) is one possible mechanism for the viral pathogenesis of asthma [3]. In addition to the focus on viral infections and asthma, an emerging body of evidence suggests that the atypical bacteria *Chlamydia pneumoniae* (Cp) and *Mycoplasma pneumoniae* (Mp) are associated with asthma [4], [5], [6], although whether these associations are causal remains a matter of some debate. Regarding Cp the preponderance of evidence favors an association with asthma severity [7]. However, there is also evidence for associations with asthma inception [8], exacerbation [9] and lung remodeling [10]. It is currently not established whether these atypical infections directly create asthma or secondarily and opportunistically infect previously susceptible asthmatic lungs [5]. In either case pathogen-specific IgE, were it to be present, might be expected to augment the severity of asthma. There have been some reports of asthma associated with specific IgE against Cp [11], [12], [13] and against Mp [14], [15] but the data are sparse. In this study we measured the prevalence of Cp-specific IgE (Cp IgE) in a sample of community adult asthma patients and investigated associations with disease severity. We also performed two case-control studies of the association of Cp IgE and asthma using healthy blood donors and non-asthmatic clinic patients as controls. Lastly, we report a preliminary exploration of whether the Cp IgE biomarker would predict treatment outcome in those subjects who elected empiric azithromycin for their asthma.

## Materials and Methods

### Ethics statement

The Dean Foundation, Madison, Wisconsin and the University of Massachusetts, Amherst, Massachusetts human subjects committees both approved the study. All clinical subjects (asthma cases and clinic controls) provided written informed consent. The St. Marys Hospital, Madison human subjects committee reviewed and approved the use of outdated and to-be-discarded Wisconsin blood donor samples in a Health Insurance Portability and Accountability Act (HIPAA) compliant fashion. The University of Massachusetts, Amherst human subjects committee likewise approved the use of Massachusetts blood donor samples. In order to comply with HIPAA standards, none of the blood donor samples could be linked to individual donors, therefore written informed consent from individuals was not obtained for these samples with the approval of the respective committees.

### Asthma subjects

One of the investigators (DLH) enrolled a consecutive series of 66 patients with physician-diagnosed asthma encountered in a community-based non-academic primary care practice. The subjects were either patients of the practice or were seen in consultation for their asthma. Asthma severity was recorded according to current guidelines as either intermittent or persistent (mild, moderate or severe) [16]. Pulmonary function test reversibility, either spontaneously or after treatment, was not a study requirement.

### Case-control study design

We performed two case-control studies. In Study 1 (blood donor controls) we tested blood donor samples for Cp IgE and compared the results to asthma cases. As controls we tested 25 platelet pheresis donor samples from Baystate Medical Center, Springfield MA and 26 platelet donors from the Red Cross and St. Marys Hospital, Madison, Wisconsin. Deidentified demographic data consisting of gender and age were available for blood donor controls. We did not test for Cp DNA in blood donor controls because these samples consisted of plasma/platelets and did not include mononuclear cells that are expected to carry this intracellular pathogen. In Study 2 (clinic controls), we tested peripheral blood samples for Cp IgE and DNA from non-asthma outpatients. These control subjects were without known acute or chronic respiratory illnesses and were matched to a case patient by gender, smoking status (current, ex-, never) and age (+/−10 years). After a case subject was enrolled, the investigator (DLH) then identified an appropriate patient who later attended the practice to be a matched control. After 20 such controls were enrolled and tested it became obvious that no significant differences would be found for Cp IgE, therefore enrollment of further clinic controls was suspended.

### Cp-specific IgE

Proteins of purified chlamydial elementary bodies (EBs) from Cp TW183 were separated on 4–20% NU-PAGE gradient gels and transferred by Western blot to PVDF membranes. Because of the low concentration of IgE in patient sera, prior to IgE blotting, sera were immunoabsorbed in an anti-human gamma-chain specific protein G agarose system to remove competing IgG as previously reported [17]. Since protein G does not bind IgE antibodies [18], [19] the resultant supernatant became ‘enriched’ for IgE. The supernatant was diluted and used to probe the individual blot strips. Blot strips were then washed and incubated with an AP-conjugated goat anti-human IgE (ε-chain specific) antibody (KPL Scientific Inc., Gaithersburg, MD). The blots were visualized after addition of BCIP/NBT alkaline phosphatase substrate solution (Sigma-Aldrich, St. Louis, MO). The identity of *Chlamydia*-specific bands recognized by patient IgE on each blot were determined based on known molecular weight patterns of the various *Chlamydia* surface proteins as previously characterized and confirmed [20], [21]. We also performed specific comparative staining with purified *Chlamydia* lipopolysaccharide (LPS) and recombinant major outer membrane protein (MOMP) to confirm identity of these proteins.

### Cp-specific DNA

PCR was performed as previously described and reported [22]. Briefly, genomic DNA was isolated from whole blood using the QIAMP DNA Blood mini kit (Qiagen Inc., Valencia, CA). Previously published Cp primers (Cpn A and B) were used to amplify a 463 bp product [23]. The PCR products were separated by gel electrophoresis on 2% agarose gels and visualized by staining with ethidium bromide. In order to independently validate the presence of Cp in our blood samples, we sent blinded samples of whole blood and extracted DNA to the lab of Dr. Bernhard Kaltenboeck (Auburn University, AL) who used the 23S rRNA gene as target as previously described for qPCR analysis [24].

### Azithromycin treatment

Some patients who were enrolled as asthma subjects in this study elected to receive empiric treatment with azithromycin from their physician for persistent and/or refractory asthma symptoms. These subjects were prescribed azithromycin 500 milligrams daily for 3 days, then 750 milligrams once weekly for an additional 11 weeks and they reported their clinical response at follow up visits as part of their usual care. A subset of these patients had serial testing performed for Cp IgE and DNA in peripheral blood.

### Statistical methods

We used Fishers Exact test for categorical variables, and t-tests for continuous variables reported as mean and standard deviation. P-values less than 0.05 (2-tailed) were considered significant.

## Results

We enrolled and tested 66 asthma patients between June 2009 and February 2011. This sample contained a wide range of ages, asthma severity and age of asthma onset. The majority of subjects were white, non-Hispanic and never and ex-smokers. Forty eight percent were encountered in consultation because they had refractory asthma. Fourteen percent reported a coexisting clinical diagnosis of COPD. Table 1 presents further details of the subject characteristics.

**Table 1:** Subject characteristics.

	Asthma cases (n = 66)	Blood donor controls (n = 51)	Clinic controls (n = 20)
Mean age (Sd)	40.9 (17.8)	49.2 (12.9)	48.6 (14.7)
Range	5–75	21–79	18–69
Gender, no. (%) male	39 (59)	34 (67)	12 (60)
Smoking, no (%)		NA	
Current	9 (14)		1 (5)
Ex-	14 (21)		5 (25)
Never	43 (65)		14 (70)
Race, no. (%)		NA	
White	57 (86)		17 (85)
Black	5 (8)		1 (5)
Asian	4 (6)		2 (10)
Mean age asthma onset (Sd)	22.4 (19.1)	-	-
<18 yoa, no, (%)	32 (48)		
≥18 yoa	34 (52)		
Asthma severity, no. (%)		**-**	**-**
Intermittent	19 (29)		
Mild persistent	6 (9)		
Moderate persistent	27 (41)		
Severe persistent	14 (21)		
Coexisitng COPD, no. (%)	10 (15)	**-**	**-**

NA = data not available; **-** = not applicable.

### Biomarker prevalence

#### Cp IgE

**Figure 1:**
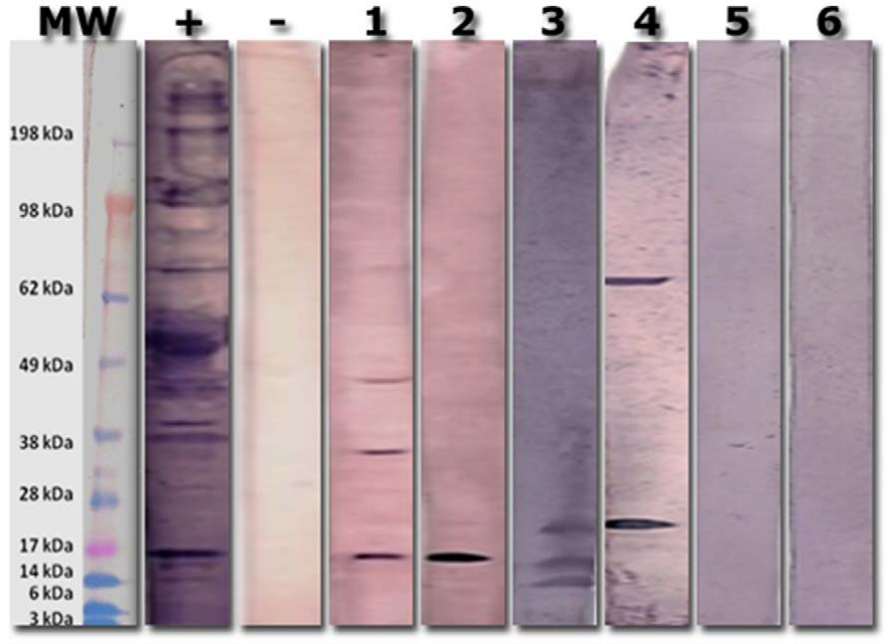
Representative Western blot for the detection of *Chlamydia*-specific IgE. Whole *C. pneumoniae* lysate was electrophoresed using SDS-PAGE and blotted onto PVDF membrane. Each patient's Cp-specific IgE antibodies were enriched by affinity chromatography and used to probe each strip. The representative figure shows the presence of *Chlamydia*-specific IgE antibodies in the serum of patients in samples 1 through 4 and absence in samples 5 and 6. The lanes labeled + and − display the respective controls.

Cp IgE was detected in 33 (50%) of the 66 asthma subjects. Figure 1 shows typical western blot strips as well as the relevant positive and negative controls. The figure clearly demonstrates that a small number of chlamydial proteins induced Cp IgE. Five known chlamydial antigens were the most commonly recognized bands: the cysteine rich protein A (CrpA, 49%), the major outer membrane protein (MOMP, 33%), the *Chlamydia* lectin binding proteins (LBP, 18, 27 and 32 KDa, 15% to 27%), the chlamydial heat shock protein 60 (cHSP60, 18%) and the chlamydial lipopolysaccharide (cLPS, 18%) (Table 2). These proteins were identified by molecular weight estimations and specific comparative staining with purified *Chlamydia* cLPS and recombinant MOMP protein as described in Methods.

**Table 2:** Cp-specific IgE band frequencies*.

Protein†	Frequency, n (%)
6 kDa LPS	6 (18)
15 kDa CrpA	16 (49)
18 kDa LBP	5 (15)
27 kDa LBP	5 (15)
32 kDa LBP	9 (27)
37 kDa SapA	2 (6)
40 kDa MOMP	11 (33)
43 kDa CHLPS	5 (15)
52–55 kDa Unknown	5 (15)
60 kDa HSP60	6 (18)
64–68 kDa Unknown	5 (15)
70 kDa HSP70	2 (6)
99 kDa Omp4	1 (3)

* = Among 33 IgE positive cases.

† = In ascending order of molecular weight.

#### Cp DNA

**Figure 2:**
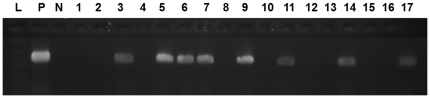
Representative PCR data. PCR products were separated on agarose gel and stained with ethidium bromide to display amplified products. The figure shows the base pair ladder (L), positive control (P), negative control (N) and representative samples with/without positive bands. Samples 3, 5–7, 9, 11, 14 and 17 are positive while the others did not display the positive band.

Cp DNA was detected in 16 (24%) of 66 asthma subjects. Figure 2 shows a representative agarose gel of individual patient sample PCR products at 463 bp. There was a strong and statistically significant association between being Cp DNA positive and being Cp IgE positive: 14 (88%) of 16 PCR positive subjects were also IgE positive (P = 0.001). In addition, there was a group of 19 (38%) of 50 PCR negative subjects who were also IgE positive, suggesting a previous infection or current persistent infection without productive progeny that could be released into the blood stream

A second lab performed qPCR on samples from 52 of our asthma cases and from 29 other samples in a blinded manner using different primers. Of the 81 total samples that were tested by both PCR methods, the second lab confirmed PCR positivity in 16 (62%) of 26 of our Cp DNA positive samples and found PCR negativity in 53 (96%) of 55 of our Cp DNA negative samples (P≤0.0001 for overall agreement by Fishers Exact test). Results were similar when the 52 asthma cases were analyzed separately: 8 (57%) of 14 of our positive samples also tested positive at the second lab, and 36 (95%) of 38 of our negative samples tested negative (P≤0.0001).

### Cp IgE association with asthma severity

**Figure 3:**
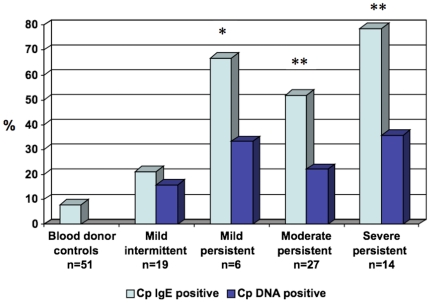
Prevalence of Cp biomarkers (IgE and DNA) according to asthma severity category. Y-axis = Percent of group testing positive. *P = 0.002 compared to control; **P = <0.0001 compared to control; P = 0.002 for linear trend in Cp IgE for asthma severity categories; P = 0.27 for linear trend in Cp DNA. Blood donor controls were not tested for Cp DNA.

Cp IgE was detected in 4 (21%) of 19 subjects with mild intermittent asthma compared to 29 (62%) of 47 with persistent asthma (P = 0.006). There was also a significant (P = 0.002) positive linear association between Cp IgE and GINA severity categories (Figure 3). Cp DNA was detected in 3 (16%) of 19 patients with mild intermittent asthma compared to 13 (28%) of 47 with persistent asthma (P = 0.36). There was no significant linear association between DNA positivity and GINA asthma severity categories (P = 0.56).

### Cp IgE association with asthma

Cp IgE was significantly (P<0.0001) more prevalent in the 66 asthma cases (33 of 66) compared to the 51 blood donor controls (4 of 51) (Table 3). Results remained significant when asthma cases were separately compared to blood donor controls from Wisconsin (1 of 26 Cp IgE positive, P<0.0001) and from Massachusetts (3 of 25 positive, P = 0.001). We found no significant differences in Cp DNA or Cp IgE prevalence between the matched cases and the matched clinic controls (Table 3).

**Table 3:** Case-Control results.

	Study 1			Study 2		
	All asthma cases	Blood donor controls	P-value*	Matched asthma cases	Matched clinic controls	P-value*
No.	66	51		20	20	
Cp IgE, no. (%)						
positive	33 (50)	4 (8)	<0.0001	11 (55)	15 (75)	0.320
negative	33 (50)	47 (92)		9 (45)	5 (25)	
Cp DNA, no. (%)						
positive	16 (24)	NA	-	8 (40)	7 (35)	1.000
negative	50 (76)	NA		12 (60)	13 (65)	

* Fishers Exact test; NA = data not available; **-** = not applicable.

### Temporal changes in Cp IgE

Nine of 66 asthma subjects had repeat Cp IgE and DNA testing performed a range of 4 to 16 months after enrolment (Table 4). Cp IgE was present at enrolment and persisted in 7 of 9 subjects and was negative at enrolment but was detected later in the other 2 subjects. Since all but one of the 9 subjects described in Table 4 received azithromycin prior to obtaining the follow up sample(s), azithromycin treatment therefore did not seem to affect detection of Cp IgE in this small sample. Cp DNA was also detected at enrolment in 7 of 9 subjects; 5 of 7 DNA positive subjects became DNA negative after azithromycin treatment. Notably in Case 6, Cp DNA reappeared in association with a relapse after azithromycin treatment. Thus, there was some indication that azithromycin treatment might diminish the ability to detect Cp DNA in peripheral blood. In Case 3, Cp DNA was not detected at baseline but was positive after treatment, despite a positive clinical response to azithromycin. In Case 9, Cp DNA detection became negative in the absence of treatment despite persistence of symptoms.

**Table 4:** Patients with multiple specimens.

Age and gender	Specimen #	Date	Cp DNA	Cp IgE	Azithromycin treatment?
1) 25 M	001	6/17/09	+	−	yes
	049	2/17/10	−	+	
					
2) 15 F	023	11/25/09	+	+	yes
	056	3/2/10	−	+	
					
3) 48 M	021	11/11/09	−	+	yes
	063	3/12/10	+	+	
					
4) 66 F	020	11/11/09	+	+	yes
	065	3/22/10	+	+	
					
5) 54 M	018	10/28/09	+	+	yes
	068	3/30/10	+	+	
	099	1/28/11	−	+	
					
6) 43 M	015	10/21/09	+	+	yes
	073	4/23/10	−	+	
	088	10/25/10	+	+	
					
7) 38 M	083	8/2/10	−	−	yes
	094	12/17/10	−	+	
					
8) 44 M	052	2/24/10	+	+	yes
	100	2/1/11	−	+	
					
9) 26 M	019	10/28/09	+	+	no
	103	2/10/11	−	+	yes

+ = detected; − = not detected.

### Cp IgE and azithromycin treatment outcome

Thirty-nine asthma patients from the cohort of 66 asthma subjects elected empiric azithromycin treatment from their personal physician and reported clinical outcome. We therefore performed an unplanned exploratory analysis of baseline Cp biomarkers and treatment outcome. Twenty-one (81%) of 26 subjects whose baseline (pre-treatment) Cp IgE was positive reported improvement compared to 12 (92%) of 13 who were Cp IgE negative at baseline (P = 0.64). Ten (71%) of 14 patients whose baseline Cp DNA test was positive reported clinical improvement versus 23 (92%) of 25 Cp DNA negative subjects (P = 0.16). Thus, neither pretreatment Cp IgE nor pretreatment Cp DNA was significantly associated with a clinical report of improvement after azithromycin treatment in these analyses.

## Discussion

We report results from asthma patients drawn from a community practice. To increase generalizability, this sample purposely included covariates such as smoking, coexisting COPD and a broad range of clinical severity. These covariates are often limited or used as exclusion criteria in asthma research [25], [26]. Significant exclusions may produce precise, but not very generalizable results. Because of our relatively small sample size, we were unable to perform meaningful subgroup analyses on smoking or on co morbid COPD. Such analyses will be important in future, larger studies because there is a growing recognition that asthma and COPD may not be entirely unrelated diseases [27]. In particular, the “overlap syndrome" (patients manifesting both asthma and COPD characteristics) appears to comprise half or more of older adults with obstructive airways disease [28]. We limited our analysis to associations with disease severity for which we had adequate power.

We detected Cp IgE in a large proportion (50%) of asthma patients from a community practice and found that Cp IgE had a positive “dose response" relationship with asthma severity. We also found a significant association between Cp IgE and an asthma diagnosis using healthy blood donors as controls. These two findings (disease association and “dose response") are two epidemiological criteria that support (but cannot in themselves prove) a causal association. Early observations led clinicians to speculate that infections, via bacterial allergy, played an important role in asthma pathogenesis [1]. Subsequent epidemiological evidence indicated that asthma was almost always associated with elevated IgE levels that were not necessarily induced by common aeroallergens [29]. This observation led the investigators to suggest that IgE associated with asthma could be related to “missing antigens." Our current findings and the research of others suggest that microbial infection could be an additional source for antigenic stimulation of IgE in asthma. This evidence has accumulated from studies showing the presence of virus- [30], *Mycoplasma*- [15] and *Chlamydia*-specific [11], [12], [13] IgE in asthma but the data are sparse. In the current study we provide further evidence that chlamydial biomarkers (Cp-specific IgE and Cp DNA) are commonly detected in outpatients with physician-diagnosed asthma.

Although *C. pneumoniae* has approximately 1000 coding genes, only a small number of antigens appeared to induce IgE production in this sample of asthmatic patients. The most commonly recognized bands of known function in our Western blot assays (CrpA, MOMP, LBPs, HSP60 and LPS) are located on the outer surface of elementary bodies and/or are secreted by the organism during acute or persistent infection. The outer membrane proteins (MOMP and CrpA) are expressed on the infectious elementary body (EB) and are therefore logical candidates as antigens to stimulate humoral immunity during the infectious phase. HSP60 is up regulated and secreted during persistent intracellular infection, and may contribute to the pathogenesis of chronic inflammatory chlamydial diseases [31]. The chlamydial LPS has been associated with acute coronary artery disease but a role in asthma has not been investigated [32]. In addition to these reasonably well characterized chlamydial antigens, we also detected 15% prevalences of IgE reactivity against two sets of chlamydial antigens of unknown function (52–55 kDa and 64–68 kDa).

We detected Cp DNA in half of subjects who were Cp IgE positive. It is possible that the remainder of Cp IgE positive, DNA negative subjects were persistently sensitized but no longer infected. Alternatively, the Cp IgE positive, DNA negative subjects might have been persistently infected but were not bacteremic at the time of sampling. Our data cannot distinguish between these possibilities. The statistically significant (P<0.01) association between Cp DNA positivity and Cp IgE positivity in peripheral blood supports the conclusion that antigenic stimulation by an active *C. pneumoniae* infection could be responsible for the presence of circulating Cp-specific IgE. Our results cannot determine whether the lung, as we suspect, was the source of the active infection. Detection of Cp DNA in peripheral blood has correlated with Cp DNA detection in BAL and bronchial biopsy specimens from subjects with COPD [33]. Our data are consistent with the possibility that a similar process occurs in asthma. Further research will be required to establish whether an active lung infection is responsible for triggering Cp IgE production in asthma, and to what extent seeding of the bloodstream from the lung can occur.

IgE positivity against one or more Cp protein antigens was significantly (P<0.001) associated with asthma severity by several criteria (Figure 3). As mentioned previously, this “dose-response" association is one epidemiological criterion supporting a causal association. Cp DNA was also more prevalent in severe persistent (36%) than in mild intermittent (16%) asthma but this difference was not statistically significant. One possible interpretation of these results is that a specific host response to *C. pneumoniae*, rather than mere presence of the organism, is required for disease expression.

In addition to an association with asthma severity, Cp IgE was also significantly (P<0.0001) associated with asthma, using blood donors as controls (Table 3). Asthma case and clinic control samples were refrigerated and shipped on ice within several days to the testing laboratory. Blood donor control samples were refrigerated until they became outdated and then shipped, raising the issue whether prolonged storage was associated with degradation of IgE resulting in lack of detection. We do not believe this is likely, however, as immunoglobulins are very stable protein molecules and refrigeration should prevent bacterial enzyme degradation or cellular uptake and processing of these Igs over this relatively short period of time [34], [35]. Somewhat surprisingly, no association of Cp IgE and asthma was found when clinic controls were examined. It is well known that hospital and clinic controls may not be representative of the general population. The higher prevalence of Cp biomarkers in non-asthma clinic controls compared to healthy blood donor controls may reflect differences in exposure to Cp infection between adults in the general population (as reflected by healthy blood donors) and in a more selected group that seeks medical care (the clinic controls). Cp IgE prevalence in clinic controls is consistent with previous data on allergen-specific IgE, in which non-allergic control groups may demonstrate differing degrees of IgE positivity [36]. The presence of specific IgE in clinic controls may indicate that other permissive factors are also required for disease expression.

Emre et al. [11] performed the only previous case-control study of the association of asthma and Cp IgE detected by immunoblotting. They compared 14 Cp culture positive children with asthma exacerbations (cases) with 3 control groups (11 Cp culture positive non-wheezing children with community acquired pneumonia, 11 culture negative pediatric asthmatics and 9 culture-negative asymptomatic pediatric non-asthmatics) and reported that 12 (86%) cases were Cp IgE positive compared to 9–22% of the control groups (P<0.01 for each case-control comparison). The most commonly recognized proteins were at 98 (82%), 78 (60%), 58–60 (71%) and 36 kDa (65%). The only commonly recognized band found in both the Emre et al. [11] study and ours was the putative chlamydial heat shock protein (HSP60, 58–60 kDa) band. Cp HSP60 secretion from infected cells can create an inflammatory response via activation of toll like receptors [37] and seroreactivity against Cp HSP60 has been associated with asthma and with airflow limitation in a previous study [38]. Both the Emre et al. [11] study and our study support a possible role for Cp IgE in asthma, and further suggest that chlamydial HSP60 should be examined further for a possible role in asthma pathogenesis.

Experimental evidence supports the possibility that the associations of Cp IgE and asthma are causal. *Chlamydia*-specific IgE production, with subsequent fixation of the IgE to FcεRI receptors on mast cells and basophils, could lead to the initiation and propagation of immediate and sustained IgE-mediated inflammation for the duration of the bacterial presence [39]. One characteristic of atypical lung infection in human asthma is the presence of increased mast cell numbers [40]. Further research is required to determine whether these infection-associated mast cells are activated by Cp IgE to produce worsening asthma symptoms. In addition, chamydial infection of dendritic lung cells has been shown to promote overall Th2 immunity and airways hyperreactivity [41], suggesting the possibility that infection might also promote a more general atopic predisposition. Recent experimental evidence indicates that chlamydial lung infection may induce or worsen several hallmarks of asthma including airway inflammation [42], [43], [44], airway hyperresponsiveness [42], [45], mucous hypersecretion [45], [46] and IL-13 production [45]. IL-13 can in turn promote susceptibility to chlamydial lung infection [47] that could in theory produce a positive feedback loop to create or worsen asthma. To this end, it is noteworthy most antigens that induce the secretion of IL-13 also stimulate IL-4 and IL-5 production. Both of these cytokines are highly pleiotrophic with multiple regulatory functions beyond IgE regulation, including an important role in modulation of macrophage activity [48]. Effects of chlamydial infection have been demonstrated in both atopic [44], [49] and non-atopic [50] animal models, and include augmented neutrophilic inflammation and Th1/Th17 effects [51]. The effects are also dependent on the timing [43], [44], [45] and the dose [44] of infection. Taken together, these experimental results coupled with the clinical observations support the possibility that human chlamydial lung infection can result in pathogen-specific IgE production that could worsen asthma, as suggested by our results. However, other mechanisms not involving IgE have also been demonstrated. These types of experimental studies may serve as platforms for elucidation of further explanatory mechanisms regarding a potential role for both pathogen-specific IgE and for other infection-related mechanisms in asthma pathogenesis.

Follow up sampling from a limited number of our subjects demonstrated clearance of Cp DNA from the circulation in 5 of 7 after treatment with azithromycin, and one of these patients had a recurrence of Cp DNA detection in the peripheral blood in association with an asthma relapse later. Due to an unplanned high frequency of patients electing empiric azithromycin treatment for their asthma, we took the opportunity to collect data on clinical response. These reports should be interpreted cautiously because they were based solely on clinical self-reports from self-selected subjects and the treatment was not blinded nor randomized. The majority of asthma patients who elected empiric azithromycin treatment for their refractory asthma reported that they experienced significant clinical improvements that were sustained after treatment was completed. These self-reports are consistent with previous data of persisting improvements in asthma symptoms after limited courses of azithromycin treatment [52], [53]. Further evidence is available from a randomized trial by Simpson et al. [54] who reported that the macrolide clarithromycin decreased airway IL-8 and neutrophilia, and improved wheezing and asthma quality of life in subjects with severe, refractory asthma who were randomized to clarithromycin.

To illustrate the importance of exploring whether biomarkers can predict treatment response in this context, we performed an unplanned exploratory analysis to test whether baseline Cp IgE was associated with patient self-reports of positive outcomes after taking azithromycin. In a previous randomized, placebo controlled trial of azithromycin in persistent adult asthma [53], Cp IgA antibodies were significantly associated with the severity of subsequent asthma symptoms. High IgA subjects compared to low IgA subjects were twice as likely to have a positive response to azithromycin, but the difference was not statistically significant, perhaps due to insufficient power. In the present study we also found no statistically significant association between baseline Cp IgE and self-reported outcome of azithromycin treatment, as over 80% of treated patients reported improvement irrespective of IgE status. These results cannot be considered conclusive because of the methodology employed, as discussed above. Nevertheless, it is worthwhile discussing these preliminary results. Assuming an actual treatment effect, the lack of predictive power for Cp IgE in our study may be explained in several ways. First, it is possible that any treatment effect was due to non-antimicrobial (anti-inflammatory or immunomodulatory) mechanisms [55] or to antimicrobial activity against other organisms [56]. Another possibility is that Cp IgE may be only one of many potential microbial mechanisms involved in “chlamydial" asthma pathogenesis. For example, *C. pneumoniae* infection of bronchial epithelial cells can release histamine in a non-allergic manner via induction of histamine decarboxylase [57]. Evidence for other potential non-allergic mechanisms of action include, but may not be limited to, generation of cytokines such as TNF-alpha, IL-8 and reactive oxygen species [58], effects on lung dendritic cells [44] and IL-17 [59], [60], immunopathologic actions of *C. pneumoniae*-specific heat shock protein 60 [38], infection-induced bronchial ciliary dysfunction [61], bronchial epithelial damage [62] and other effects on lung structure [45].

In summary, *Chlamydia pneumoniae*-specific IgE was commonly detected by immunoblotting in a consecutive series of outpatients with asthma, and was significantly and positively associated with worse disease severity and with asthma using healthy blood donors as controls. These results are compatible with the concept that organism-specific IgE generated and sustained by *C. pneumoniae* chronic infection may be one of several mechanisms that contribute to asthma pathogenesis. Our results support the inclusion of Cp IgE as a biomarker in future studies investigating a potential role for microbial infection in asthma.
